# Mathematical models for lymphatic filariasis transmission and control: Challenges and prospects

**DOI:** 10.1186/1756-3305-1-2

**Published:** 2008-02-12

**Authors:** Subramanian Swaminathan, Pani P Subash, Ravi Rengachari, Krishnamoorthy Kaliannagounder, Das K Pradeep

**Affiliations:** 1 Vector Control Research Centre, Indira Nagar, Medical Complex, Pondicherry – 605 006, India

## Abstract

**Background:**

Mathematical models developed for describing the dynamics of transmission, infection, disease and control of lymphatic filariasis (LF) gained momentum following the 1997 World Health Assembly resolution and the launching of the Global Programme to Eliminate Lymphatic Filariasis (GPELF) in 2000. Model applications could provide valuable inputs for making decisions while implementing large scale programmes. However these models need to be evaluated at different epidemiological settings for optimization and fine-tuning with new knowledge and understanding on infection/disease dynamics.

**Discussion:**

EPIFIL and LYMFASIM are the two mathematical simulation models currently available for lymphatic filariasis transmission and control. Both models have been used for prediction and evaluation of control programmes under research settings. Their widespread application in evaluating large-scale elimination programmes warrants validation of assumptions governing the dynamics of infection and disease in different epidemiological settings. Furthermore, the predictive power of the models for decision support can be enhanced by generating knowledge on some important issues that pose challenges and incorporating such knowledge into the models. We highlight factors related to the efficacy of the drugs of choice, their mode of action, and the possibility that drug resistance may develop; the role of vector-parasite combinations; the magnitude of transmission thresholds; host-parasite interactions and their effects on the dynamics of infection and immunity; parasite biology, and progression to LF-associated disease.

**Summary:**

The two mathematical models developed offer potential decision making tools for transmission and control of LF. In view of the goals of the GPELF, the predictive power of these models needs to be enhanced for their wide-spread application in large scale programmes. Assimilation and translation of new information into the models is a continuous process for which generation of new knowledge on a number of uncertainties is required. Particularly, a better understanding of the role of immune mechanisms in regulating infection and disease, the (direct or immune mediated) mode of action of current drugs, their effect on adult worms, their efficacy after repeated treatment, and the population genetics of drug resistance are important factors that could make the models more robust in their predictions of the impact of programmes to eliminate LF. However, if these models are to be user-friendly in the hands of programme managers (and not remain as research tools), it would be necessary to identify those factors which can be considered as the minimum necessary inputs/outputs in operational settings for easy evaluation and on-site decision making.

## Background

Two mathematical simulation models, EPIFIL (population based, deterministic) and LYMFASIM (individual based, stochastic), have been developed at the Vector Control Research Centre, Pondicherry, respectively in collaboration with the Wellcome Trust Centre for the Epidemiology of Infectious Disease, Department of Zoology, University of Oxford, Oxford and the Department of Public Health, Erasmus University, Rotterdam, for tracking the dynamics of lymphatic filariasis (LF) transmission and control [1,2]. Model parameters were estimated using data from an integrated vector management programme in Pondicherry, India [1,3]. Both models have been used to predict the long-term impact of control programmes (mass chemotherapy/vector control) and assess the prospects of elimination [1,4-6]. This assumes significance in the context of the Global Programme to Eliminate Lymphatic Filariasis (GPELF), with Mass Drug Administration (MDA).

Recently Stolk et al[7] compared EPIFIL and LYMFASIM in terms of their structure and parameter quantifications and highlighted deficiencies that impede their wide spread application for decision support. These authors found that, despite differences in model structure and parameterization, both models were able to predict the duration of control required for elimination under assumptions based on current biological understanding. However, actual predictions varied between models that were due to the choice of criteria used in the models for elimination: EPIFIL assumes that transmission will cease to occur when the microfilarial (mf)-prevalence drops (deterministically) below 0.5% following MDA, whereas LYMFASIM considers the likelihood (in a number of stochastic simulations) of reaching zero prevalence within 40 years after the start of MDA. However, when similar assumptions were used, the predictions by both models were similar in terms of required coverage and duration of MDA. In this communication, we discuss additional factors which are crucial for predictions by models, and the need of extensions to enhance their predictive power for decision support.

## Discussion

### Drug-related factors

#### Mode of action of drugs

Currently available anti-filarial drugs (diethylcarbamazine – DEC, albendazole and ivermectin) differ in their mode of action. Available evidence suggests that ivermectin and albendazole have direct killing effect on microfilariae (mf) whereas DEC acts through immune mechanisms [8]. The heterogeneity in response to DEC [9] also indicates that DEC does not kill the parasite directly. In addition, the immune-mediated effect is expected to last longer, which may influence model predictions. Estimates from a model based on analysis of data from a hospital trial showed that a single dose DEC (6 mg/kg) can reduce the mf-production of adult worms by 67% at 12 months post-treatment and 87% at 24 months post-treatment, and reduce the mf-density in the peripheral blood by 57% at 12 months and 52% at 24 months post-treatment [9,10]. The increasing effect of DEC on mf-production up to 24 months post-treatment is suggestive of slow immune mediated damaging effect of DEC on adult worms, which occurs at an extended time scale. However, reliable efficacy estimates, at least for DEC, need to be obtained through meta-analysis by generating data from clinical trials with different periods of follow-up. Also, models should account for the heterogeneity in drug response by individuals as well as for the average duration of the drug effect ('effective duration'). In LYMFASIM the heterogeneity in drug-response by individuals and the 'effective duration', measured as the period during which the female parasite recovers from treatment, are considered as stochastic variables. However, only the former is being used for model prediction.

#### Microfilaricidal effect of drugs

Since efficacy estimates (measured as percent reduction of mf load with respect to baseline) vary between clinical trials, the most reliable estimates are based on rigorous meta-analysis of data from randomized controlled trials. Such estimates are available for ivermectin [11] and also for combination therapies of albendazole with DEC or ivermectin, albeit based on a limited number of trials [12]. Similar analyses for DEC and albendazole monotherapy may be useful to more confidently predict the synergistic effect of combination therapy, and determine its superiority over a single dose regimen.

#### Macrofilaricidal effect of drugs

DEC is known to have also a macrofilaricidal effect, *i.e*., to be effective against adult worms [13-15]. There are similar indications for albendazole alone [16] or in combination with DEC [17-19]. However, ivermectin seems to have no macrofilaricidal effect [20], although model based estimates of its effect on mf-production by adult worms suggests that it may have a damaging effect on the reproductive system of female worms [9,11]. In the absence of diagnostic tools to measure adult worm burden accurately, only indirect estimates have been used in both models [4-7]. This uncertainty will have greater impact on the predicted durations of MDA for LF elimination given the fact that the average and distribution of adult worms' life expectancy has a large influential effect on the population dynamics [21].

#### Effects of drug combinations

When co-administration of albendazole with either DEC or ivermectin was initially proposed, albendazole was not intended or expected to enhance the efficacy of either DEC or ivermectin. However, limited trials with the recommended combination of albendazole with ivermectin suggest that co-administration of ivermectin with albendazole is superior to either of the two drugs given alone. In the case of DEC co-administered with albendazole, the results have not been so equivocal, although the combination therapy appears to have an edge over either drug given alone [22]. While additional studies may be useful to reliably obtain efficacy estimates as well as to determine the superiority of the combination, if any, current policies are clearly in favour of the combination.

#### Repopulation of blood by mf after anti-fecundity effects

Repopulation of the blood by mf depends on the effects of anti-filarial drugs on host immunity and worm fecundity. Drugs can improve host immunological responsiveness without affecting the viability and fecundity of adult worms (DEC [8]; ivermectin [23]). This in turn can clear mf during repeated treatments and thus reduce microfilaraemia. Drugs may also express their action in different ways such as sterilizing, killing or affecting the fecundity of the adult worms. These effects can affect the repopulation of mf in the blood. Anti-fecundity effects may be manifested in two ways: (i) by an overall reduction in reproductive capacity across all worms, or (ii) by sterilization (temporary or permanent) of a proportion of the female worms. Temporarily sterilized worms may resume their reproduction and release mf after a period of recovery. Both these effects may vary between individual worms and therefore may be stochastic in nature. That anti-fecundity effects may vary between worms has been incorporated into LYMFASIM, but its implications have not yet been fully explored or understood.

#### Change in efficacy after repeated treatment

**Figure 1:**
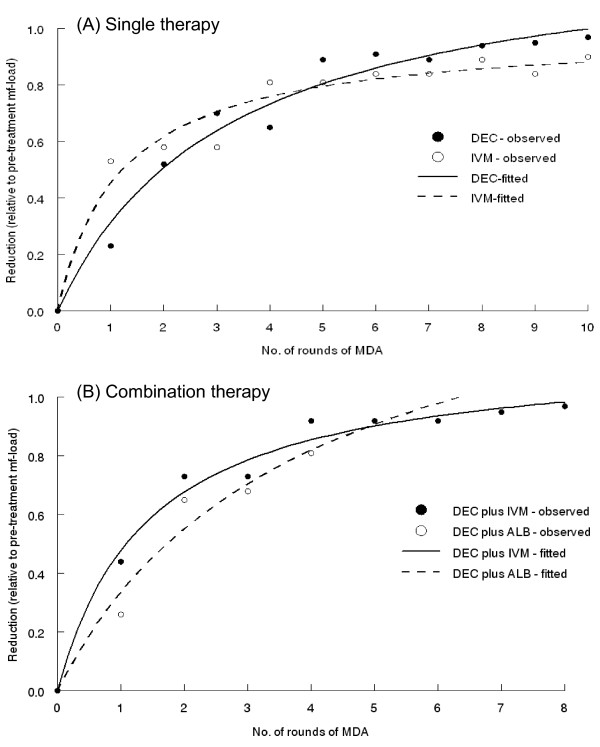
Reduction in microfilaraemia load (efficacy) following repeated treatments with single (A) or combination therapies (B) with antifilarial drugs. Data are from filed trials in India [31]. Dots are observations and the lines are based on the fitting of non-linear function of the form y=ax(1+bx) with *y *being the efficacy after *x *number of treatments. The parameter *a *measures the initial rate of increase of *y *with *x*, and *y *reaches a saturation level *b'*, with b'=bc.

Clinical trials of single (DEC or ivermectin) or combination (albendazole plus DEC or ivermectin) drug therapies are limited to provide estimates for the change in efficacy (measured as percent reduction of mf load with respect to baseline) after repeated treatments. However, available trial results suggest that efficacy of single drug therapy (DEC or ivermectin) increases with multiple single dose treatments at a constant rate and reaches a plateau after a few treatments [24,25]. This effect does not necessarily imply that the drug has become less efficacious but that the percent reduction in microfilaraemia effected by treatment may also depend on mf load at the point of treatment, with the highest reductions recorded for heavier microfilaraemia, i.e., microfilaricidal efficacy declines with decreasing mf load (density-dependent efficacy). (For an individual, whose initial mf load is 100, a decrease to 5 following treatment would mean a reduction by 95%; whereas for a person with 5 mf ending up with 1 mf, the reduction will be of the order of 80%). Such density-dependent efficacy is also apparent from a study in which four repeated full (each with 6 mg/kg for 12 days) courses of treatments were given over a period of one year: efficacy did not vary among those who received one (90.4%), two (95.7%), three (97.4%) and four (100%) courses of treatment. However, those who required at least 2 courses of treatment, had a higher pre-treatment mf-density than those who needed only one-course (6 mg/kg × 12 days) of treatment [26]. Similar observations are also evident from community based trials with single dose DEC (6 mg/kg) administered monthly [27,28], or semi-annually [29] for one year and annually for four years [30] and DEC (6 mg/kg) or ivermectin (400 μg/kg) for 10 years (Fig. 1A[31]).

The efficacy of co-administration of albendazole (400 mg) with DEC (6 mg/kg), or ivermectin (150 μg/kg) is constant and independent of the number of repeated treatments (albendazole plus DEC: 92%, 95%, 98% and 94% following, respectively, the first, second, third, and fourth treatments [19]; albendazole plus ivermectin: 87% and 93% following, respectively, the first and second treatments [32]) *i.e*. the efficacy is maximum upon first treatment, decreasing afterwards with the increasing number of treatments and decreasing mf-load (density-dependent efficacy). The results of field trials with repeated administration of DEC plus ivermectin or DEC plus albendazole are also suggestive of an initial constant rate of increase and decline after few repeated treatments (>90% reduction in each of four annual rounds of MDA with DEC plus ivermectin [30]; 70–96% reduction over four rounds of MDA with DEC plus albendazole [33]; Fig. 1B[31]).

The currently available predictions of the prospects of elimination by both EPIFIL and LYMFASIM are based on the assumption that single drug efficacy (DEC, ivermectin, and albendazole) is constant and independent of the number of repeated treatments [4,5,34]. But with the evidence of change in efficacy after repeated treatments with single or combination therapies, as well as the consideration that drug resistance may emerge (see section on population genetics of drug resistance below), the chemotherapy components of the models need to be updated as density-dependent processes, relaxed by the effects of interventions, can considerably alter the prospects of elimination [35,36].

#### Population genetics of drug resistance

Drug resistance is one of the causes for treatment failure in the control of many parasitic diseases. Large scale MDA with ivermectin in areas of the Onchocerciasis Control Programme in West Africa has raised concerns about the possibility that individuals who have been repeatedly treated may exhibit suboptimal responses to ivermectin (their rate of skin repopulation by mf of *Onchocerca volvulus *is faster than expected [37,38]). In populations of *Wuchereria bancrofti *in Ghana, the frequency of a phenylalanine to tyrosine change at position 200 of the beta-tubulin gene (TYR200, a marker for resistance to benzimidazoles in veterinary nematodes), was found to be significantly higher in microfilariae obtained from patients treated once or twice with albendazole plus ivermectin than in mf from those untreated by the GPELF [39], whereas, analysis of *W. bancrofti *populations in India revealed absence of this genetic change, suggesting albendazole susceptibility [40]. These results indicate that selection for albendazole and ivermectin resistance may already be occurring in some localities. Though strong evidence for resistance to DEC is lacking [41,42], more studies are required to understand whether or not selection under chemotherapeutic pressure is indeed occurring, to elucidate the possible mechanisms of resistance, as well as to measure the frequency of putative resistance markers in a broad spectrum of epidemiological settings for sound incorporation of worm genetic heterogeneity with respect to drug susceptibility in the models. Recently, EPIFIL has been modified to account for parasite population genetics, and has been used to examine the consequences of a hypothetical spread of benzimidazole resistance on the impact of MDA with albendazole in combination with ivermectin or DEC [43], or the impact of multidrug resistance (to ivermectin and albendazole) under various assumptions of the genetics of drug resistance [44]. The LYMFASIM model can also be extended in a similar way; its microsimulation approach may prove to be more effective for accounting of any genetic variability between individual parasites within hosts that may be identified by molecular genetics to improve its predictive value.

#### Drug distribution and consumption

Programmes report only coverage of drug distribution; no data on actual consumption of the drug are currently available. So far both models consider coverage as a proxy for the actual consumption, but there are considerable gaps between coverage (measured as the proportion of the population that received the drug) and consumption (measured as the proportion of the population that actually consumed the drug) [45]. Quantification of their relationship [46] and its incorporation into models would enhance the robustness of model predictions.

### Reproductive biology and mating success

Reproductive lifespan, worm mating, its modality and frequency; the per worm rate of mf production (worm fecundity and fertility rates), and longevity of mf in the blood are parameters governing the reproductive biology of the parasite. The values of these parameters may vary between worms and/or mf according to a (largely unknown) probability distribution. In addition, worm survival and fecundity may be age-dependent. No direct estimates are available for the means and uncertainty of all these parameters. Schulz Key [47] has studied the reproductive biology of *Onchocerca volvulus *and assessed the fecundity of the parasite (through the examination of embryograms of adult worms excised from palpable, subcutaneous nodules), whereas such studies are not possible for LF (given the difficulty in obtaining adult worms from the lymphatics). However, indirect estimates of some of the biological characteristics of *W. bancrofti *have been made in LF (longevity; mf production per female worm [3,48]). Unfortunately, the lack of appropriate animal models in which to mimic the natural parasite dynamics and behaviour has hindered the study of biological features that are relevant for models (but see [49]). In light of such uncertainties, sensitivity analysis of the models to plausible variation of model parameter values will be useful to ascertain the extent to which such variations may influence model predictions and their usefulness for decision making. Incorporation of stochastic effects of these biological parameters into the model is necessary to derive more realistic quantitative conclusions. Since EPIFIL is a population based deterministic macro-simulation model, it does not consider detailed aspects of the reproductive biology of the parasite, whereas LYMFASIM being an individual based, micro-simulation model, can more easily handle such biological complexities of the parasite. For example the number of adult male and female worms is a determinant of the probability of mating and production of mf. Thus LYMFASIM has the potential to strengthen its biological validity and to extend its predictive power to determining transmission thresholds and breakpoints and their associated uncertainty.

### Role of vector-parasite combinations

Parasite persistence and its elimination/eradication, besides heterogeneities in human and vector processes, are also strongly influenced by host-parasite interactions [36]. Three types of possible processes have been recognized through the analysis of developmental transitions within filarial vector-parasite combinations: (i) proportionality (describing a number of infective larvae, L3, developing per mosquito which increases linearly with mf intake at a constant rate, therefore revealing lack of regulation or density dependence), (ii) facilitation (the per microfilaria rate of development into L3 larvae per mosquito increases with mf intake) and (iii) limitation (the per microfilaria rate of development into L3 decreases with mf intake, describing a relationship in which the number of L3 developing within the mosquito increases with mf intake in a saturating fashion until it reaches a plateau at high mf intakes) [50]. The operation and strength of any of these processes depend on the specific vector parasite combination: limitation is found to occur in *Culex *[50-54] and *Aedes *[51,54] mosquitoes transmitting *W. bancrofti*; facilitation in *Anopheles *vectors [50,51,55,56] also transmitting *W. bancrofti*, and proportionality has been observed in *Mansonia *mosquitoes transmitting *Brugia malayi *[51]. It has been proposed that elimination/eradication of infection would require more interventional efforts in places where vectors exhibiting limitation or proportionality patterns prevail, because the transmission thresholds for these processes would be much lower compared to those in settings with vectors exhibiting facilitation (but see [57,58]). Existence of ecological differences in vector-parasite relationships is reported to be one of the reasons for inconsistent patterns in the success of large scale control programmes in different parts of the world (Melanesian islands [59]; China [60]; Pacific islands [56]). Therefore models should be specifically calibrated for each vector-parasite combination and for each ecological setting. This means that although 'global, mainly qualitative trends' (*i.e*., outcomes from strategic models) may be useful advocacy tools, detailed, and mainly quantitative predictions (from tactical models) will be needed to guide locale-specific LF policy.

### Magnitude of transmission thresholds

The decision to stop/continue MDA depends on the magnitude of thresholds for transmission success ('transmission threshold') and parasite establishment/maintenance ('breakpoint density') in the human and vector hosts. These thresholds are critical minimum values (of vector biting rate, of parasite density) derived from the transmission processes relating vector-to-host and host-to vector below which the parasite population would not be able to persist [21]. Both types of threshold are expected to vary with exposure to vector biting, the eliciting of protective immune responses, and to depend on the regulatory processes that occur within vector-parasite combinations (among other factors). The transmission threshold is defined as the vector density below which infection establishment and sustained transmission in the population would not occur (the infection would not become endemic). Breakpoint densities refer to parasite densities below which infection cannot persist [21,36]. A relatively simple population dynamic model has been used to determine the transmission threshold(s) [34]. In this simple model, the authors related mf intensity or prevalence in a community as a function of annual vector biting rate and parameters governing regulation of parasite populations in the human and vector hosts. Nevertheless, the estimates indicate not only the existence of thresholds for filariasis elimination programmes but also suggest that there are significant vector-parasite differences for these thresholds. Reliable estimates are crucial to make credible predictions of the optimal intervention strategies and their impact. This requires detailed modeling of the density-dependent processes operating within human and vector hosts and on the heterogeneity between persons with regard to exposure to vector biting, parasite aggregation and the acquisition of immune responses [61,62]. Although the LYMFASIM simulation model has included density-dependent processes and heterogeneities [2], the model should be tested in different endemic settings so that it can be used more effectively to obtain reliable estimates of region-specific transmission thresholds.

### Infection and disease dynamics related factors

#### Infection dynamics

**Figure 2:**
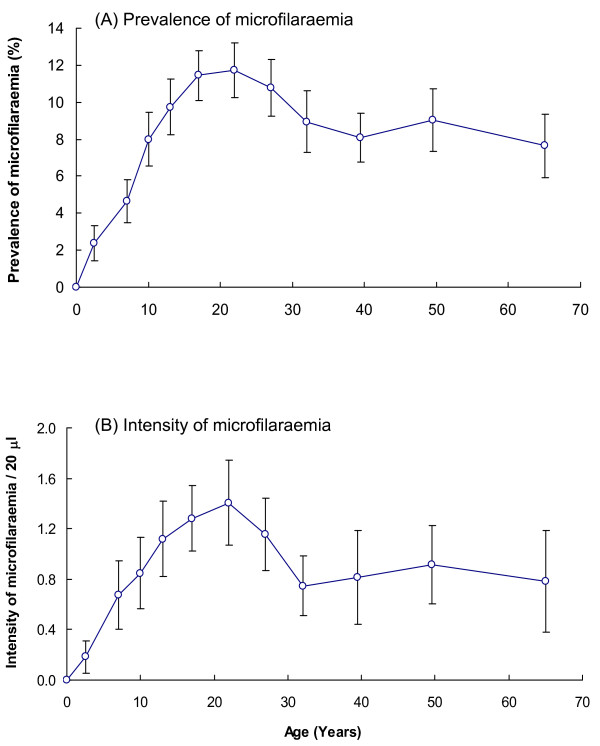
Age related prevalence (A) and intensity (B) of *Wuchereria bancrofti *microfilarial load in Pondicherry, India [66]. Error bars are 95% confidence intervals for the observed prevalence and intensity of microfilariae.

A better understanding of the processes regulating infection in the human host is essential for predicting the long-term impact of MDA and assessing the prospects of elimination. Age-infection (prevalence and intensity) profiles derived from cross-sectional surveys have been used to interpret the processes that shape the age-infection profiles. Four types of age-intensity profiles can emerge from cross-sectional data [63]. The shape of age-infection profiles is determined by the following processes: (i) age-dependent exposure, (ii) parasite-induced host mortality, (iii) heterogeneity within the host population, (iv) clumped infection, (v) density-dependent parasite mortality, and (vi) density-dependent parasite establishment. (If age profiles are investigated by using indirect measures of infection, which they usually are, *i.e*., microfilariae rather than adult worms profiles, then density-dependent female fecundity will also be important). Increase in infection (mf) prevalence/intensity with age either with (linear-saturating) or without (linear) reaching a plateau at adult age suggests that acquired immunity may not be very important, whereas an increase with age to reach a peak followed by a decline (convex pattern) suggests the operation of either acquired immunity or a reduction in parasite acquisition as a consequence of decreasing exposure with increasing age [62,63]. The former also being accompanied by a decrease in the age at which peak infection is reached (or peak shift [64]). Application of both EPIFIL and LYMFASIM models to Pondicherry data and EPIFIL to East African data showed that the models had to include acquired immunity to explain the observed convexity in the age-infection profiles of *W. bancrofti *mf, although there is indication of transmission dependency [65]. Despite strong indication for the role of acquired immunity from Pondicherry (Fig. 2[3,66]) and East Africa [65], a recent meta-analysis of age-prevalence data from India and African countries casts doubts over the role of immune mechanisms in regulating LF infection in humans [67]. The impact of this uncertainty on the predicted duration of control would have serious implications on the decision to stop/continue MDA programmes. Premature cessation of MDA programmes may reduce the ability of developing an immune response and thus increase the likelihood of resurgence. This will be further worsened if drugs do not act on adult worms or their productivity. Therefore both EPIFIL and LYMFASIM models need to be validated in different epidemiological settings to explore the plausible mechanisms/processes regulating infections in humans.

#### Disease dynamics

At present both EPIFIL and LYMFASIM models have considered very simple assumptions of disease dynamics [1,2]. Fits of the EPIFIL model to the Pondicherry situation indicated that disease progression (lymphoedema/hydrocoele) is a consequence of worm burden, but not necessarily associated with immunopathological mechanisms. However, available evidence suggests that, although progression to hydrocoele may be associated with worm burden, progression to lymphoedema is due to immunopathological reactions and secondary bacterial infections [68]. Further progression of lymphoedema depends on the frequency of acute episodes of adenolymphangitis (ADL), and vice versa [69], and therefore models should also consider other modalities of disease progression. LYMFASIM should be extended to include the relationship between infection and disease, and the estimation of these parameters for quantification and validation. Prevention of disease incidence can be a long term outcome of GPELF with the goal of elimination of LF as public health problem. It would also be important to assess cost and cost-effectiveness of MDA for disease prevention, and of morbidity management (hydrocoelectomy/limb care) for disability prevention.

## Summary

• LF has been targeted for global elimination by 2020. Mathematical models could be potential tools for decision-making if they are able to reliably predict the impact of control programmes/interventions.

• Current models need to be improved to enable them to make more realistic predictions. This will necessitate validation in different epidemiological settings and incorporation of new knowledge.

• Presently available simulation models should account for the mode of action of drugs (*e.g*. direct effects or immune mediated effects), drug resistance, and changes in efficacy after repeated treatment. This necessitates generation of knowledge on the effects of drugs on adult worms.

• Parasite-related factors such as its reproductive biology need to be quantified as they are critical for predictions based on models.

• Models should be fine-tuned to different epidemiological settings to explore the mechanisms regulating infection and disease.

• Incorporation of knowledge on disease dynamics and progression can help set new targets to reach the goal of LF elimination as a public health problem, or identify aspects that would need to be addressed to achieve this target.

• There is an urgent need to consolidate and bring out user friendly models with the minimum necessary inputs/outputs for application in decision making and evaluation by programme mangers. Models will remain only research tools if their scope of application in GPELF is not broadened.

## Competing interests

The author(s) declare that they have no competing interests.

## Authors' contributions

SS conceived the main points presented in the manuscript and drafted the original manuscript. SPP and KK provided unpublished evidence to support the main points presented in the manuscript. SPP, RR and KK provided significant intellectual input into framing the major points presented. PKD provided intellectual input into the arguments presented. All authors read and approved the final manuscript.
